# Immune microenvironment heterogeneity characterizes biologically distinct KRAS^mut^/SPOP^mut^ and KRAS^mut^/PIK3CA^mut^ mesonephric-like adenocarcinoma subtypes revealed by integrated whole-exome and transcriptomic profiling

**DOI:** 10.3389/fimmu.2025.1605227

**Published:** 2025-07-16

**Authors:** Jing Zeng, Qingli Li, Kemin Li, Lu Yang, Lian Xu, Wei Wang, Kaixuan Yang, Qingbo Wei, Jin Wang, Changbin Zhu, Rutie Yin

**Affiliations:** ^1^ Department of Obstetrics and Gynecology, West China Second University Hospital of Sichuan University, Chengdu, Sichuan, China; ^2^ Key Laboratory of Birth Defects and Related Diseases of Women and Children, Sichuan University, Ministry of Education, Chengdu, Sichuan, China; ^3^ Department of Pathology, West China Second University Hospital of Sichuan University, Chengdu, China; ^4^ Department of Translational Medicine, Amoy Diagnostics Co., Ltd, Fujian, Xiamen, China

**Keywords:** mesonephric-like adenocarcinoma, immune microenvironment, molecular biology, whole-exome, transcriptomic profiling gynecological mesonephric-like adenocarcinoma, kidney development, tumor microenvironment, KRAS

## Abstract

**Objective:**

This study aims to uncover the molecular biology and immune microenvironment of gynecological mesonephric-like adenocarcinoma (MLA).

**Methods:**

To determine the comprehensive characteristics of MLA, 17 patients with MLA were retrospectively enrolled in this study. Whole-exome sequencing and mRNA sequencing were performed to explore the molecular features. The biological differences between MLAs and epithelial-initiated gynecologic tumors reported in The Cancer Genome Atlas database were also analyzed.

**Results:**

*KRAS* mutations (82.4%) were considered the driving mechanism and were co-mutated with PIK3CA (47.1%) and SPOP (23.5%), but their functions were mutually exclusive. In addition, pathways and genes associated with kidney development were upregulated in MLA patients. Compared with adjacent tissues and common gynecological tumors in The Cancer Genome Atlas, Th2 signature and resting mast cells account for the majority in MLAs, rendering an immunosuppressive TME. Particularly, the expression levels of IFNG, IFN6, and IFN1 KRAS_SPOP group, significantly lower than the rates found in KRAS_PIK3CA group. *KRAS*_*SPOP mutant* MLAs, exhibited reduced immune infiltration in their tumor microenvironment.

**Conclusion:**

This is the first study to demonstrate the comprehensive molecular characteristics of MLA and detect biologically distinct subtypes of *KRAS* ^mut^/*SPOP* ^mut^ and *KRAS* ^mut^/*PIK3CA* ^mut^ MLAs.

## Introduction

Mesonephric adenocarcinoma (MA) is a rare malignant tumor of the female reproductive tract (1). It typically occurs in the cervix, originating from the remnants of the mesonephric duct, and is often associated with mesonephric duct hyperplasia (2). Mesonephric-like adenocarcinoma (MLA) arises in atypical mesonephric duct remnants, is not linked to mesonephric duct hyperplasia, and histologically resembles MA (3). MLA was first reported by McFarland et al. in 2016 as a rare gynecological tumor; moreover, it is included in the WHO Classification of Tumours Editorial Board (88) classification of tumors of the female reproductive system (4, 5). MLA most commonly occurs in the uterus, followed by the ovaries, and is rarely found in the fallopian tubes, mesocolon, or urinary tract (6, 7). The majority of adenocarcinomas with mesonephroid features occur in the uterus (74.7%, 115/154), with a smaller number of cases reported in the ovary (25.3%, 39/154) (8). Owing to its high invasiveness, MLA is often diagnosed at International Federation of Gynecology and Obstetrics (FIGO) stage II–IV and is prone to early recurrence and distant metastasis (9, 10). Compared with endometrioid adenocarcinoma, patients with uterine MLA have a lower progression-free survival (PFS) (11). Moreover, they have a poor prognosis, with 60%–80% of them experiencing recurrence or death. The most common site of distant metastasis is the lung, followed by the liver. Patients with ovarian MLA have a tumor-free survival of 24.5 months, PFS of 68%, and overall survival (OS) of 71% (12).

MLA exhibits morphological features similar to MA, with various growth patterns, including tubular, glandular, papillary, reticular, glomerular, and solid patterns, with lumens containing colloid-like eosinophilic material (13, 14). Histopathologically, MLA tissues exhibit mixed morphological features following hematoxylin and eosin staining. The MLA tissues are negative or show limited positivity for estrogen receptor staining; positive for TTF-1, CD10, and GATA-3 staining in most cases; and positive for calretinin staining in some cases (15, 16). Recurrent *KRAS* mutations, microsatellite stability, and frequent gains of chromosome 1q are observed in MLA (17, 18). Recent studies have shown that *KRAS* mutation is a unique molecular feature of uterine and ovarian MLAs, suggesting that this mutation is involved in the occurrence and development of MLA (13, 19). *KRAS* activating mutations are the most common molecular alterations in middle renal cell carcinoma, leading to sustained activation of mitogen-activated protein kinase and subsequent activation of multiple downstream targets (20). Most MLAs lack *TP53* mutations and POLE exonuclease domain hotspot mutations and are negative for mismatch repair genes (21). Patients with MLA often have gene mutations associated with endometrioid tumors, such as K*RAS(90%), PIK3CA(28%), PTEN (23.1%)* and *CTNNB1(14%)* mutations, along with some copy number variations (18, 22). The PTEN-PI3K-AKT pathway is frequently altered in gynecological tumors, especially in endometrial cancer, where nearly half of the patients have PIK3CA mutations (23). Multiple studies have detected SPOP mutations in the MLA (24, 25). The mutation frequencies of SPOP in ovarian and endometrial mesonephric-like tumors are 27% and 8% respectively (18). The gene encoding the E3 ubiquitin ligase substrate-binding adaptor SPOP is frequently mutated in endometrial cancer (EC), and it is also one of the factors driving the progression of EC (26). Both MLA and MA exhibit moderate levels of genomic instability, defined as copy number variations of whole chromosomes or long/short arms of chromosomes (18). Among these, 1q, Chr10, and Chr12 are the most frequently amplified segments, and 1p is the most frequently lost segment (18). Gene mutation is an important topic of research in life sciences, and detection methods have been rapidly developed. Detecting gene mutations aids in the early diagnosis and treatment of diseases (27). In addition, tumor immune microenvironment (TIME) has been reported to be associated with tumor prognosis and immunotherapy benefits in many cancers (28). However, the molecular pathology and tumor immune microenvironment research of MLA is still in its infancy. Exploring the molecular and TME characteristics of MLA will help develop more treatment options.

Next-generation sequencing (NGS) can effectively capture extensive genomic information on tumorigenesis, progression, and biological behavior (29). In the new era of precision medicine, NGS has become a valuable tool for tumor diagnosis and treatment. It provides personalized treatment for patients through in-depth analysis of the genetic characteristics of tumors (30). Owing to the low incidence of MLAs, research on this type of tumor is still lacking, especially with regard to molecular biology and the immune microenvironment. In this study, integrated DNA- and RNA-level analysis of MLA was performed to explore the molecular features, immune microenvironment, and differences between MLAs and other gynecologic tumors.

## Materials and methods

### Enrolled samples and detection methods

This study retrospectively analyzed the medical records of patients with MLA admitted to the West China Second University Hospital between January 1, 2010 and December 30, 2022. A total of 18 cases of gynecological MLAs (from 3 different sites) were included. Total DNA and RNA were extracted from formalin-fixed and paraffin-embedded (FFPE) tumor and peritumoral specimens. The AmoyDx Panoramic View^®^ Tumor Gene Detection Kit (AmoyDx, xiamen, China) and AmoyDx^®^ Human Transcriptional Gene Detection Kit (AmoyDx, xiamen, China) were used for WES and RNA sequencing to analyze gene mutations and molecular features of the tumors. This study was approved by the Ethics Committee of the West China Second University Hospital.

### WES and RNA sequencing

To perform NGS, DNA and RNA were extracted from FFPE samples using AmoyDx^®^MagPure FFPE DNA LQ Kit and AmoyDx^®^ FFPE RNA Extraction Kit, respectively, following the manufacturer’s instructions. xGen^®^ Exome Research Panel v1 (IDT:1056115) was used to construct DNA libraries. The collected products were amplified and quantified using KAPA Hotstart Ready Mix and Qubit. The size of the library was determined using an Agilent 2100 bioanalyzer. After pooling, libraries were sequenced at 2 × 150 bp for end reads using Novaseq6000. The sequencing data were analyzed and annotated using ANDAS. Sequencing data were cleaned by removing adapters and low-quality reads (quality <15) or poly N and then aligned to the human reference genome version 19 (hg19). PCR repeats were tagged and eliminated. The final VCF files were generated by comparing indels and nucleotide polymorphisms. The single nucleotide variants and indels were further filtered using the following criteria: (i) at least ≥5 readings supporting the variant and ≥5% variant allele frequencies supporting the variant; (ii) population frequency of >2% in 1000g, ExAC, or GnomAD database; (iii) if the variant is not located in the CDS region; and (iv) if variants are not annotated as (likely/predicted) carcinogenic in the OncoKB database. These filtered variations were functional and available for further data analysis. RNA sequencing was performed using Novaseq6000. Genome mapping of each sample’s reads was performed using a transcriptome constructed from GRCh37/hg19 using STAR 2.7, and transcript abundances were measured in transcripts per million using RSD v1.3.3.

### CNV analysis

The Genomic Identification of Significant Targets in Cancer (GISTIC2.0, version 2.0.23) algorithm was employed to investigate the prominent regions of somatic copy number alterations (31). GISTIC2.0 was used with specific parameters, including -ta 0.8, -td 0.8, -genegistic 1, -smallmem 1, -broad 0, -brlen 0.98, -conf 0.99, -armpeel 1, -savegene 1, and -gcm mean.

### Mutation signatures

The deconstructSigs (version 1.8.0) R package was used to calculate the mutational signatures of gene mutations obtained from WES data (32). Signatures 1 to 30 from the COSMIC database were obtained for this analysis (https://cancer.sanger.ac.uk/signatures/signatures_v2/). Somatic single nucleotide variants and small insertions and deletions were considered.

### Differential gene expression and functional enrichment analysis

The transcripts per million matrix was subjected to a log2 transformation and was then quantile-normalized using the preprocess Core R package (version 1.56.0). The limma R package (version 3.50.0) was utilized to identify DEGs with the set criteria of a *p*-value of <0.05 and an absolute log2 fold-change of >1. Subsequently, DEG enrichment and GSEA were performed using the clusterProfiler R package (version 4.2.2) with an adjusted *p*-value threshold of 0.05. The gene sets for the enrichment analyses were derived from the Gene Ontology, Kyoto Encyclopedia of Genes and Genomes, HALLMARK, and Reactome databases within the Molecular Signatures Database (33, 34).

### Published dataset

In this study, the gene expression matrices for tumor samples from TCGA for TCGA–ovarian cancer (n = 210), TCGA–uterine corpus endometrial carcinoma (n = 549), and the “Adenocarcinoma” subtype within TCGA–cervical squamous cell carcinoma and endocervical adenocarcinoma (n = 48) were obtained. These matrices were also subjected to log2 transformation and quantile normalization.

### Signature analysis of the TME and markers related to cell proliferation

The TME was evaluated by calculating various TME-related signatures using the single-sample GSEA method via the GSVA R package (version 1.42.0). The signatures included the Functional Gene Expression signature, a collection of 28 immune gene sets, and Danaher signature (28, 35, 36). Additionally, CIBERSORT was utilized to assess immune cell infiltrations using the leukocyte gene signature matrix LM22, and 1,000 permutations were performed to estimate the relative abundance of immune cells (37). For proliferation assessment, multiple signatures were employed via single-sample GSEA: CINSARC (38), Core ESC-like Module (39), Sixteen_Kinase (40), and GGI (41).

### Quantification and statistical analysis

The Mann–Whitney U test was used to compare continuous variables, whereas the Fisher’s exact test was used to compare discrete categorical variables. Survival curves were constructed using the Kaplan–Meier estimator and then compared using the log-rank test. To evaluate the predictive performance of PFS, time-dependent receiver operating characteristic curve analyses were performed. Additionally, Cox proportional hazards regression analysis was used to determine hazard ratios along with their 95% CIs. All statistical analyses were conducted using R version 4.1.2 and its associated packages.

## Results

### Baseline characteristics of the enrolled patients

**Figure 1:**
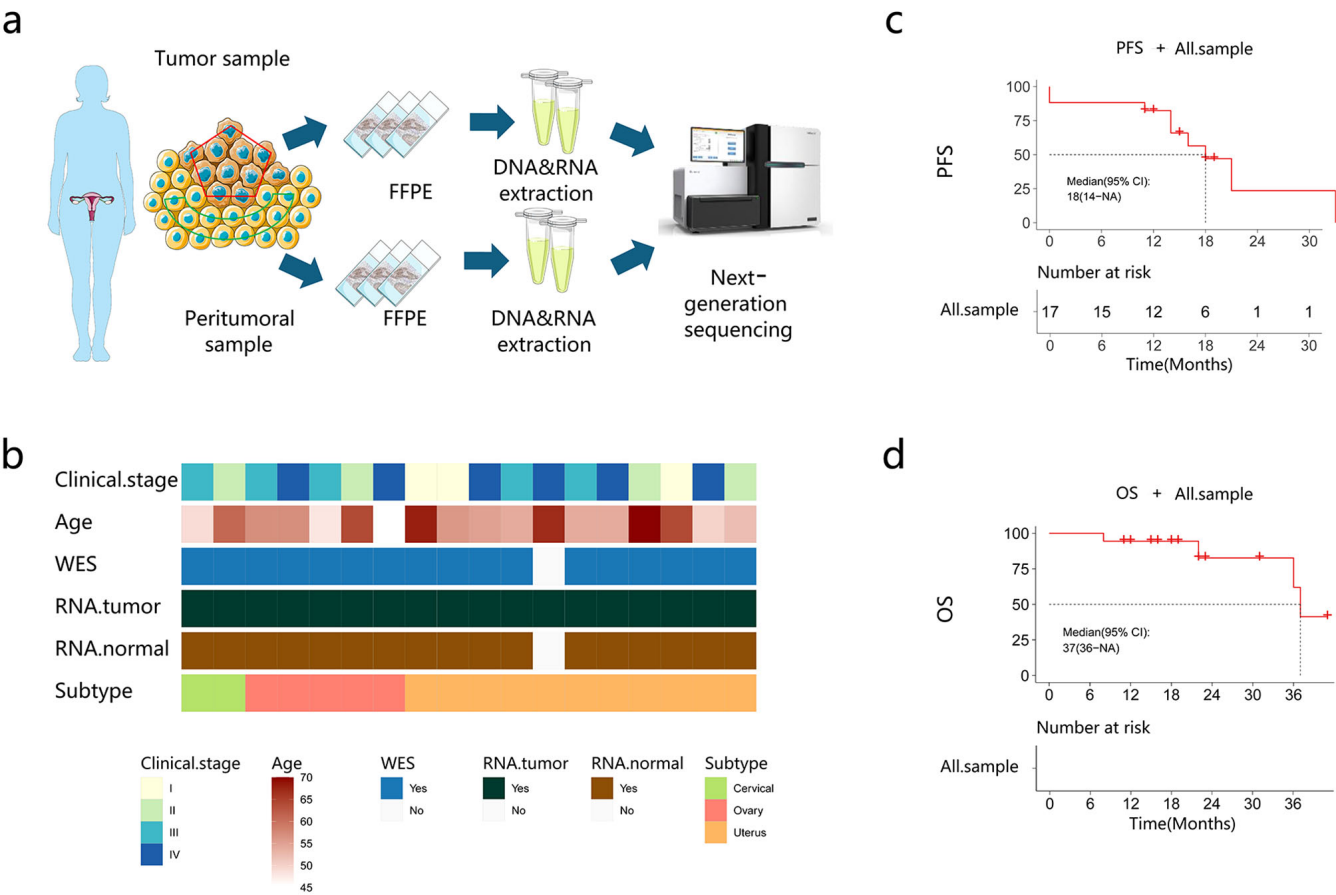
Baseline characteristics of the patients. **(a)** The study flow chart. **(b)** Patient clinical data. **(c, d)** Progression-free survival (PFS) and overall survival (OS) of the recruited patients.

The clinicopathological features of 18 patients with gynecological MLA are shown in **Supplementary Table 1**. A representative pathological diagnosis based on immunohistochemistry is depicted in **Supplementary Figure 1**. The median patient age was 56 (45–70) years. The initial stage at diagnosis ranged from IA to IVB. Surgical procedures included bilateral salpingo-oophorectomy, omentectomy, and appendicectomy. Pelvic lymph node dissection and para-aortic lymph node dissection were performed if necessary. Postoperative therapy included chemotherapy (14/18), radiation therapy (8/18), targeted therapy (3/18), and immunotherapy (1/18). The median follow-up time was 18.5 (95% confidence interval [CI], 8.26–28.74) months. This study performed whole-exome sequencing (WES) and mRNA sequencing in patients with MLA at three sites (cervix, n = 2; ovary, n = 5; uterus, n = 10) (**Figures 1a, b**). The median PFS and OS were 14.5 (95% CI, 8.08–20.9) and 18.5 (95% CI, 8.3–28.7) months, respectively (**Figures 1c, d**).

### Mutation landscape of MLA

**Figure 2:**
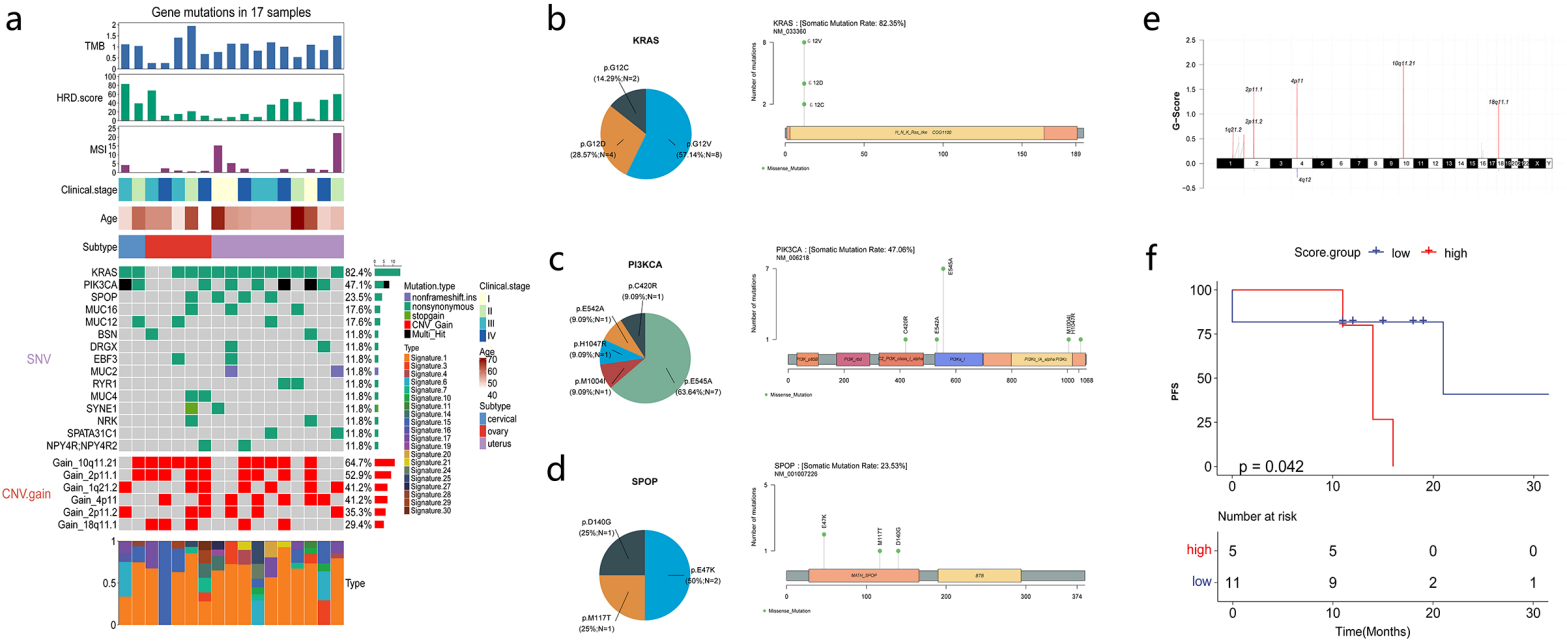
Mesonephric-like adenocarcinoma (MLA) mutations and molecular factors associated with survival. **(a)** Mutation profiles of MLA samples. **(b–d)** Proportion and schematic of mutation sites in *KRAS*, *PIK3CA*, and *SPOP*. **(e)** Schematic of chromosomal copy number variations. **(f)** Correlation analysis between Signature 15 and progression-free survival (PFS).

WES was performed in 18 patients, and only 17 patients whose sequencing data passed quality control were subsequently analyzed (**Figure 2a**). A total of 575 somatic nonsynonymous alterations were identified. WES of patients’ samples revealed low tumor mutation burden and homologous recombination deficiency scores. *KRAS* mutations were detected most frequently (82.4%, 14/17), followed by *PIK3CA* (47.1%) and *SPOP* (23.5%) mutations, which were mutually exclusive to each other. The median tumor mutation burden was 1.03 mut/Mb (0.26–1.94). The median homologous recombination deficiency score was 21 (4–83). The median microsatellite instability (MSI) score was 1.36% (0%–22.22%). COSMIC signature analysis identified an age-related signature 1 as a universal feature of MLA. The mutation sites for *KRAS*, *PIK3CA*, and *SPOP* are shown in **Figures 2b–d**. *KRAS* mutation sites involved the G12 region, including G12C, G12V, and G12D. The main *PIK3CA* mutation site was E545A, followed by C420R, E542A, H1047R, and M1004I. *SPOP* mutation sites included E47K, M117T, and D140G. CNV analysis revealed amplifications at chromosomes 10q11.12, 4q11, 2q11, and 18q11.1, and no common tumor-associated driver or suppressor genes were observed (**Figures 2a, e**).

The correlation of patients’ clinical characteristics and gene expression with PFS and OS was analyzed. Patients’ age, MSI status, homologous recombination deficiency score, tumor mutation burden, and clinical stage were not associated with PFS and OS (**Supplementary Figures 2a, b**). The correlation between COSMIC signatures and PFS revealed that patients with high signature 15 scores had shorter PFS (**Figure 2f**).

### Significantly upregulated genes and pathway analysis in MLA

**Figure 3:**
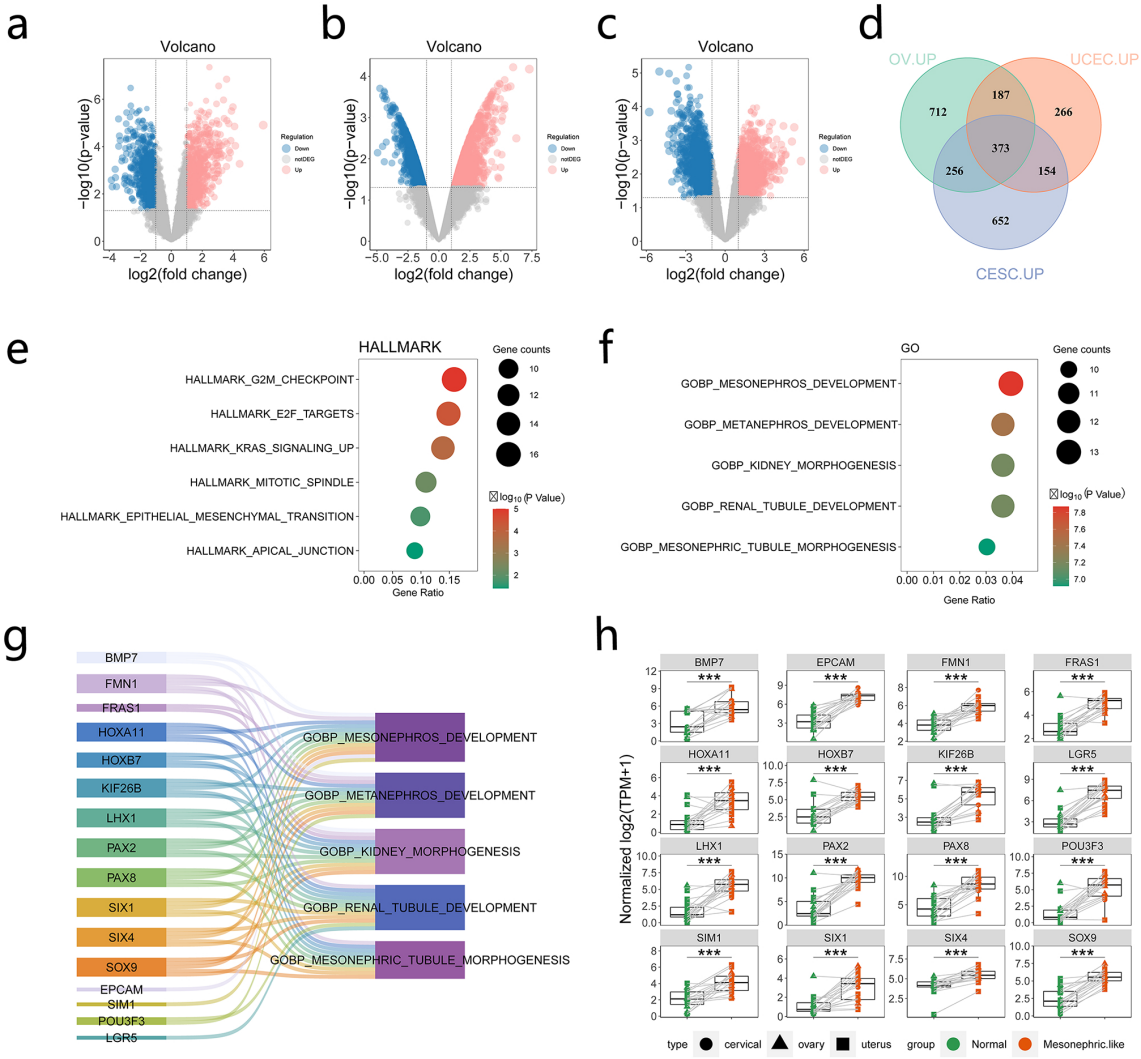
Genes and pathways significantly upregulated in mesonephric-like adenocarcinoma (MLA). **(a–c)** Comparison of upregulated and downregulated genes in uterine, cervical, and ovarian MLA samples compared with normal samples (red dots indicate upregulated genes, whereas blue dots denote downregulated genes). **(d)** Intersection of upregulated genes in MLA at three different sites. **(e)** Kyoto Encyclopedia of Genes and Genome (KEGG) enrichment analysis of upregulated genes. **(f)** Gene Ontology (GO) enrichment analysis of upregulated genes. **(g, h)** Genes in pathways related to kidney development based on GO enrichment analysis. ***: P ≤ 0.001.

Differentially expressed genes (DEGs) between MLA and paired normal samples were analyzed to explore pathways associated with MLA. The volcano plot demonstrated significant DEGs between MLA samples (uterus, cervix, and ovary) and adjacent tissues (**Figures 3a–c**) (**Supplementary Table 2**). Principal component analysis demonstrated that mesonephroid carcinomas of different origins did not cluster in terms of either mRNA expression (**Supplementary Figure 3a**) or single-sample gene set enrichment analysis (GSEA) (Functional Gene Expression and HALLMARK pathway analysis) (**Supplementary Figures 3b, c**) of related genes. These results suggested that mesonephroid carcinomas from the three sites have similar expression profiles and signaling pathways, which can be combined and analyzed as a whole. This study intersected the upregulated genes of MLAs at the three sites and found 373 upregulated genes (**Figure 3d**). These genes were subjected to Gene Ontology and Kyoto Encyclopedia of Genes and Genomes pathway enrichment analyses. Kyoto Encyclopedia of Genes and Genome analysis revealed that these genes were mainly enriched in G2M checkpoint, E2F target, and KRAS signaling up (**Figure 3e**). Gene Ontology analysis showed that the upregulated genes were mainly related to biological processes associated with kidney development, including mesonephros development, metanephros development, kidney morphogenesis, renal tubule development, and mesonephric tubule morphogenesis (**Figure 3f**). Sixteen genes (*BMP7, FMN1, FRAS1, HOXA11, HOXB7, KIF26B, LHX1, PAX2, PAX8, SIX1, SIX4, SOX9, EPCAM, SIM1, POU3F3, LGR5*) were involved in these biological processes, and the expression levels of these genes were significantly upregulated in MLAs (**Figures 3g, h**). These results suggest that the upregulated genes in MLAs are mainly involved in cell cycle regulation and kidney development processes.

### Comparison of immune microenvironment characteristics of MLAs and normal tissues

**Figure 4:**
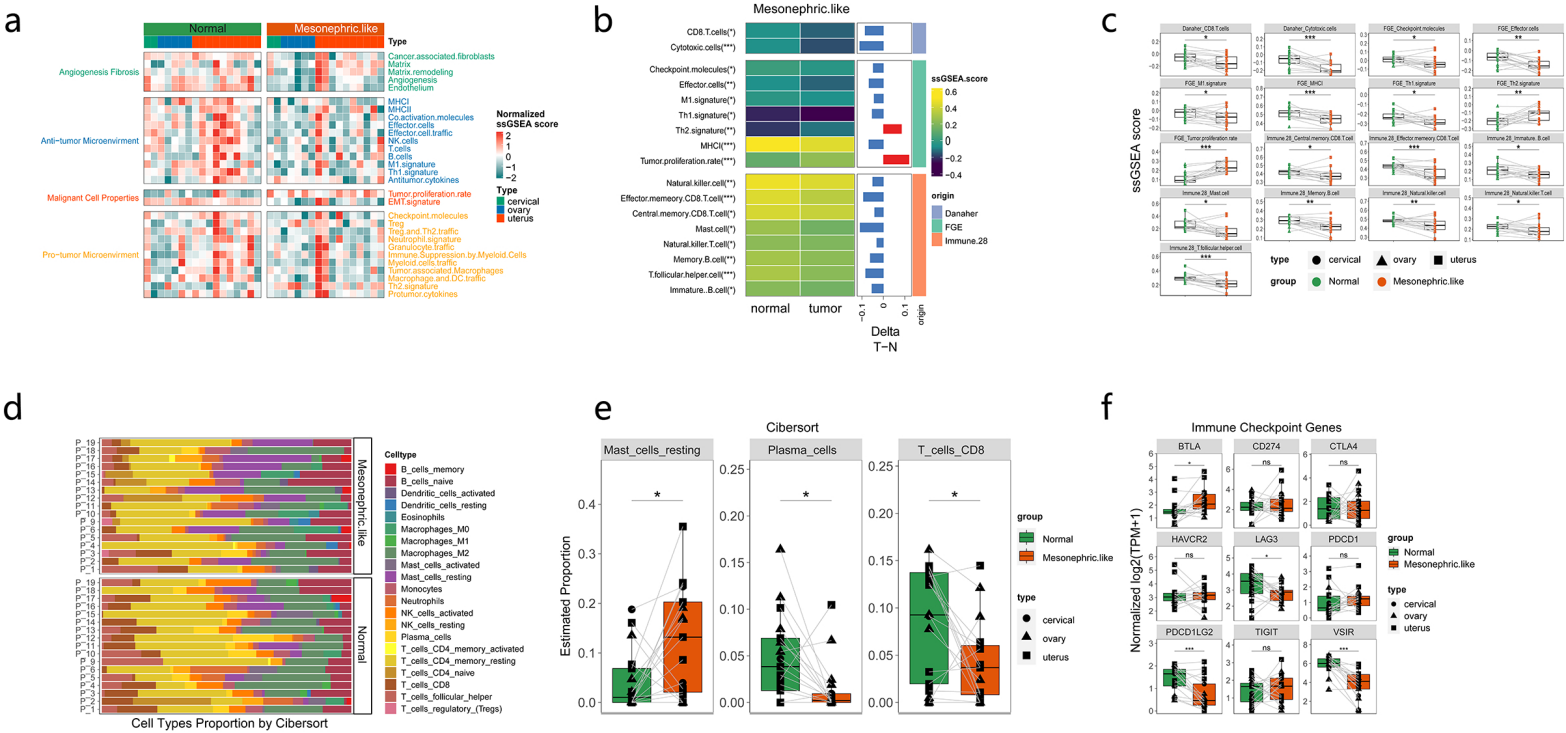
Immune microenvironment in mesonephric-like adenocarcinoma (MLA). **(a)** Differential expression of related factors in pathways closely related to tumorigenesis or immune activation. **(b-d)** Comparison of immune-infiltrating cells in MLA vs. normal tissues. **(e)** Differential expression of immune-related cells between MLAs from three sites and adjacent tissues. **(f)** Comparison of the expression of immune checkpoint–related genes between MLAs at three sites and normal tissues. ns: not significant (P > 0.05); *: P ≤ 0.05; **: P ≤ 0.01; ***: P ≤ 0.001.

The immune-related signatures and proportions of immune-infiltrating cells were compared between MLAs and adjacent tissues to evaluate the immune microenvironment characteristics of MLAs. Angiogenesis, fibrosis, and antitumor immunity were downregulated, whereas tumor proliferation was upregulated in MLAs (**Figure 4a**). We also analyzed the differences in the expression of fibroblast markers FAP and MFAP5 in MLA and normal tissues. The fibroblast marker FAP and MFAP5 were significantly upregulated in MLA compared to normal tissues (**Supplementary Figure 4**). GSEA revealed that various immune-related signatures, such as CD8 T cells, MHC, natural killer cells, and effector cells, were significantly downregulated in MLAs (**Figures 4b, c**). The proportion of immune-infiltrating cells in MLAs differed from that in normal tissues (**Figure 4d**). CIBERSORT analysis showed that the proportion of resting mast cells increased, whereas the proportions of plasma cells and CD8 T cells decreased in MLAs compared with those in normal tissues (**Figure 4e**). The differences in the expression levels of immune checkpoint–related genes between MLAs and normal tissues were analyzed. The research results showed that in the MLAs group, the expression of BTLA significantly increased, while the expressions of LAG3, PDCD1LG2 and VSIR decreased significantly. (**Figure 4f**). The differential expression of these immune checkpoints in MLAs and three types of gynecologic tumors (ovarian cancer, cervical squamous cell carcinoma and endocervical adenocarcinoma, and uterine corpus endometrial carcinoma) in the TCGA database was also analyzed (**Supplementary Figure 5**). These findings revealed that MLAs had immunosuppressive characteristics.

We also analyzed the differences in immune characteristics between KRAS mutant MLA and KRAS mutant endometrial cancer in TCGA. KRAS mutant MLA exhibits a more suppressive TME than that of KRAS mutant UCEC. CD4+ cells, CD8+ T cells, NK cells, and M1 macrophages, dendritic cells, are significantly underexpressed in MLA tumors (P < 0.05), indicating lower immune activation. Conversely, M2 macrophages, fibroblasts, and regulatory T cells (Tregs), Th2/17 cells which related to immune suppression, are highly expressed in MLA tumors (P < 0.05), reflecting a strong immunosuppressive tumor microenvironment (**Supplementary Figure 6**).

### Renal development–related pathways and genes were significantly upregulated in MLAs

**Figure 5:**
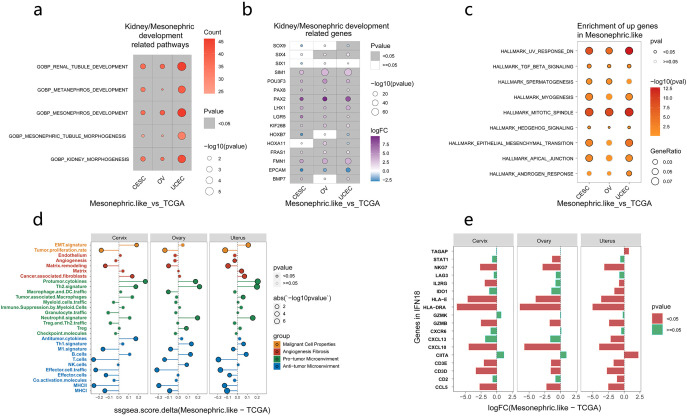
Expression of pathways and genes related to kidney development in mesonephric-like adenocarcinomas (MLA) vs. nonmesonephric-like gynecologic tumors. **(a)** Pathways related to kidney/mesonephric development in MLAs. **(b)** Genes associated with kidney/mesonephric development in MLAs. **(c)** Pathways significantly upregulated in MLA vs. nonmesonephric-like gynecologic tumors. **(d)** Differences in immune microenvironment–related factors between MLA and nonmesonephric-like gynecologic tumors (the abscissa denotes the difference between the MLA score and TCGA score; if > 0, the MLA score is higher, and if < 0, the TCGA score is higher). **(e)** Expression of IFN18 pathway genes in MLA, with green indicating significant results and red representing insignificant results).

The differential expression of kidney/mesonephric development pathways and genes between our MLA samples and nonmesonephric-like gynecologic tumors in TCGA database was analyzed. The results showed that pathways related to kidney/mesonephric development were significantly upregulated in MLAs compared with those in nonmesonephric-like gynecologic tumors (**Figure 5a**) (**Supplementary Table 3**). Similarly, genes related to kidney/mesonephric development (*SIM1*, *PAX2*, *PAX8*, *FMN1*) were significantly upregulated in MLAs (*p* < 0.05) (**Figure 5b**). Gene Ontology enrichment analysis revealed key genes involved in mesonephric tubule morphogenesis in MLAs (**Supplementary Figures 7a–c**). *PAX2*, *PAX8*, and *LHX1* were enriched in MLAs obtained from the cervix, ovary, and uterus, consistent with the classic pathological immunohistochemistry results of MLA. Differences in signaling pathways and immune microenvironment factors between our MLA samples and nonmesonephric-like gynecologic tumors were analyzed. Significantly upregulated pathways in MLAs included TGF-β signaling, myogenesis, mitotic spindle, and mesenchymal transition (**Figure 5c**) (**Supplementary Table 4**). Immune microenvironment analysis showed higher immune scores in nonmesonephric-like gynecologic tumors in the cervix, ovary, and uterus (**Figure 5d**). Differences in immune-related signatures between MLAs and gynecologic tumors (uterine corpus endometrial carcinoma, cervical squamous cell carcinoma and endocervical adenocarcinoma, and ovarian cancer) were analyzed (**Supplementary Figure 8**). MLAs showed lower scores for effector cell traffic, M1 signature, MHCI, MHCII, and T cells and higher scores for Th1 and Th2 signatures compared with conventional gynecological tumors, indicating a lack of antitumor immune environment in MLAs. The expression levels of IFN18 pathway genes, which are positively correlated with immunity in MLAs, were also analyzed. The results showed that most of these genes were not significantly expressed in MLAs (**Figure 5e**). These findings imply that kidney/mesonephric development pathways and genes are upregulated in MLAs, and the tumor microenvironment (TME) is immunosuppressed.

### Differences in pathways, immune characteristics and prognosis between *KRAS_SPOP* and *KRAS_PIK3CA* mutation groups in patients with MLAs

**Figure 6:**
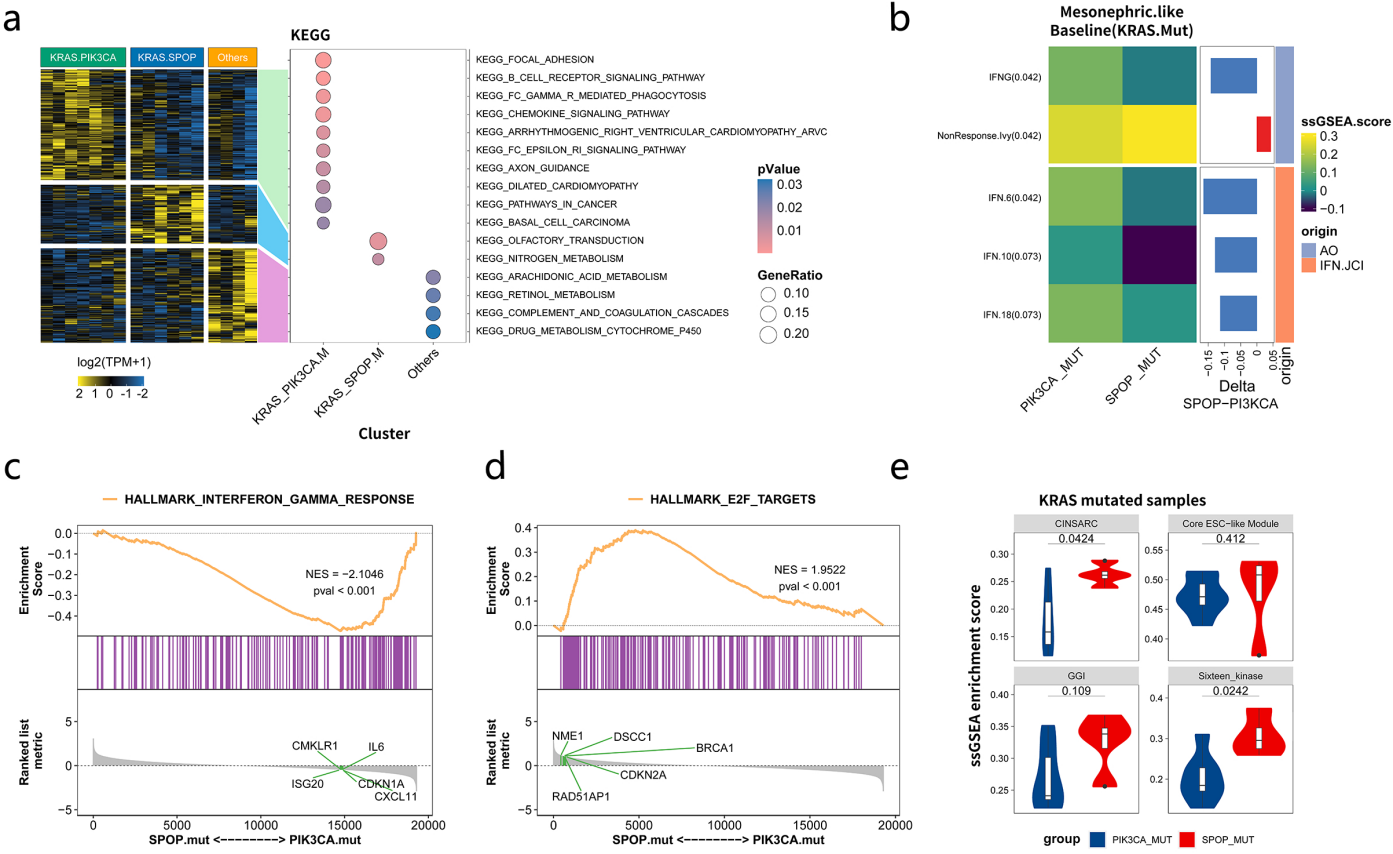
Differences in immunological characteristics among the KRAS_PIK3CA, KRAS_SPOP, and other groups in patients with mesonephric-like adenocarcinomas (MLA). **(a)** Pathways enriched by mutated genes in KRAS_PIK3CA, KRAS_SPOP, and other groups. **(b)** Differences in the immune microenvironment between KRAS_SPOP and KRAS_PIK3CA groups. **(c, d)** Major pathways enriched in KRAS_SPOP and KRAS_PIK3CA groups. **(e)** Differential expression of markers related to cell proliferation (Complexity Index in Sarcomas [CINSARC], Core ESC-like Module, genomic grade index [GGI], and Sixteen_kinase) in KRAS_SPOP and KRAS_PIK3CA groups. Sixteen_kinase refers to the kinase score of 16 genes encoding serine/threonine kinases involved in mitosis.

As commutations of *KRAS/SPOP* and *KRAS/PIK3CA* were observed in most patients and were mutually exclusive to each other, subgroup analyses were performed among KRAS_SPOP, KRAS_PIK3CA, and other groups (no commutations between *KRAS*, *PIK3CA*, and *SPOP*). Enrichment analyses (**Figure 6a**) showed that genes in the KRAS_PIK3CA group were mainly enriched in focal adhesion, B cell receptor, and chemokine signaling pathways. Genes in the KRAS_SPOP group were enriched in olfactory transduction and nitrogen metabolism. Differences in immune signatures among the three groups were analyzed. The results indicated that the KRAS_PIK3CA group had a more active immune status than the KRAS_SPOP group (**Supplementary Figure 9a**). The expression levels of *IFNG*, *IFN6*, and *IFN18* were higher in the *KRAS*_*PIK3CA* group than in the *KRAS*_*SPOP* group (**Figure 6b**) (**Supplementary Figure 9b**). The interferon-γ pathway was enriched in the *PIK3CA*-comutated group, and the E2F target pathway was enriched in the *SPOP*-commutated group (**Figures 6c, d**). The expression levels of markers related to cell proliferation (Complexity Index in Sarcomas [CINSARC], Core ESC-like Module, genomic grade index [GGI], and Sixteen_kinase [kinase score of 16 genes encoding serine/threonine kinases involved in mitosis]) in *KRAS_SPOP* and *KRAS*_*PIK3CA* groups were analyzed. GSEA revealed that the expression levels of CINSARC, GGI, and other markers related to cell proliferation were higher in the *KRAS*_*SPOP* group (**Figure 6e**), indicating that the KRAS_*SPOP* group had higher cell proliferation and malignancy than the KRAS_*PIK3CA* group. The expression levels of CINSARC and Sixteen_kinase in the *KRAS_SPOP* group were higher than those in the *KRAS_PIK3CA* group (**Supplementary Figure 9c**). These results revealed that *SPOP* and *PIK3CA* mutations involve different pathways and immune microenvironments in patients with *KRAS*-mutated MLA. In patients with MLA, *PIK3CA* mutation promotes immune regulation, whereas *SPOP* mutation promotes cell proliferation.

We also analyzed the differences in PFS and OS between the KRAS^mut^/PIK3CA^mut^ group and the KRAS^mut^/SPOP^mut^ group. The results showed that there were no significant differences in PFS (P=0.781) and OS (P=0.154) between the two groups (**Supplementary Figure 10**).

## Discussion

MLAs are rare tumors with limited treatment options (21). To develop potential therapeutic strategies of MLAs, further exploration of its pathogenesis is warranted. Complex cellular interactions within the TME play a central role in cancer progression, affecting tumor initiation, growth, invasion, therapeutic response, and drug resistance (42). A deeper understanding of tumor molecular features and the TME will lead to the development of innovative therapeutic strategies (43).Through integrated WES and transcriptomic profiling, we comprehensively characterized the distinct molecular profiles and TME of MLAs, aiming to establish a molecular foundation for improved diagnostic strategies and targeted therapeutic interventions.

According to a previous study, targeted sequencing based on NGS revealed systemic mutations in MLAs (18). However, this study provided more comprehensive information on molecular mutations for MLA detection based on WES. Our results showed that *KRAS* mutations were detected most frequently (82.4%), followed by *SPOP* and *PIK3CA* mutations, consistent with previous research (7, 16, 21). The KRAS signaling pathway was enriched in DEGs between MLAs and normal tissues. These findings suggest that *KRAS* mutation is a dominant driver of MLAs. In the current study, *KRAS* mutation sites included G12C, G12V, and G12D. The Food and Drug Administration has approved two *KRAS* G12C inhibitors-sotorasib and adagrasib—as treatment options for *KRAS* G12C-mutated nonsmall-cell lung cancer (44–46). Recently, the Food and Drug Administration approved adagrasib plus cetuximab for previously treated patients with *KRAS* G12C-mutated colorectal cancer (47). Adagrasib showed encouraging clinical activity and was well tolerated among patients with *KRAS* G12C-mutated solid tumors (48). *KRAS* G12D is a promising therapeutic target, but no approved inhibitors are currently available. The *KRAS* G12D inhibitors MRTX1133 and HRS-4642 have shown antitumor activity in preclinical and early clinical studies (49, 50). Research on pan-*KRAS* inhibitors is still in its early stages. Several pan-*KRAS* inhibitors (e.g., RMC-6236 and BI-2865) are being investigated at preclinical or early clinical stages worldwide (51, 52). Recently, *PIK3CA* mutation-targeting drugs have become a new focus in the treatment of lung, breast, and other cancers. Alpelisib has been approved by the Food and Drug Administration for treating hormone receptor–positive, HER2-negative, *PIK3CA*-comutated advanced or metastatic breast cancer (53, 54). These studies also provide direction for targeted therapy in patients with *KRAS*-mutated MLAs.

The mitogen-activated protein kinase signaling pathway mutations are highly prevalent in MA/MLA (21). Our study identified additional pathways that may be involved in MLA progression. This study showed that upregulated genes in MLA were mainly enriched in G2M_checkpoint, E2F_Target, and KRAS signaling pathways. The G2M checkpoint pathway is a key mechanism regulating cell cycle progression, especially the transition from G2 to mitosis phase (M phase) (55). Mutations in genes involved in the G2M checkpoint pathway can disrupt its function (56), leading to uncontrolled cell cycle progression and allowing cells with damaged DNA to proliferate. E2F plays a key role in determining the timing of cell division. The expression levels of E2F target genes gradually increase during G1 phase and reach critical levels to allow cells to pass through the restriction point and enter S phase (57). Interestingly, 16 genes (*BMP7, FMN1, FRAS1, HOXA11, HOXB7, KIF26B, LHX1, PAX2, PAX8, SIX1, SIX4, SOX9, EPCAM, SIM1, POU3F3, LGR5*) were associated with kidney development. *HOXA11* and *HOXB7* are typically highly expressed during the segmentation formation and organ differentiation stages of the embryo from the 3rd to the 5th week (58). *PAX2* and *PAX8* play a crucial role in the embryonic development from the 4th to the 6th week and in the organogenesis of the eyes, inner ears, kidneys and thyroid glands, with higher expression during development (59). *SIX1* and *SIX4* play important roles in the sensory organ and muscle development during the 5th to 7th week of the embryo (60). *SOX9* is highly expressed during the cartilage formation and gonad differentiation stages of the embryo from the 6th to the 8th week (61). *EPCAM* is mainly expressed during the epithelial cell differentiation process from the 4th to the 6th week of the embryo (62). *FRAS1* is highly expressed during the formation of the basement membrane, skin and kidney development during the 5th to 7th week of the embryo (63). *HOXA11, HOXB7, PAX2/8, SIX1, LGR5, EPCAM*, and *SOX9* are typical oncofetal genes that are reactivated in carcinogenesis (64–69). SIM1 is essential for the development and function of hypothalamic paraventricular neurons and is also expressed in the kidney and muscles (70). The molecular mechanism by which SIM1 regulates MLA progression requires further exploration. FMN1 variants have been linked to the occurrence of colorectal cancer, glioma, and pancreatic cancer (71, 72). Mechanistically, FMN1 promotes strong mechanical cohesion, leading to highly invasive motility (73). PAX2 is required for the mesenchymal–epithelial transformation of the intermediate mesoderm into kidney and Mullerian duct epithelial structures, including the fallopian tubes, uterus, and vagina (74). PAX2 is the most sensitive and specific marker that can distinguish MAs from ovarian endometrioid carcinoma and can be used as a first-line marker with ER/PR and GATA3/TTF1 (75). PAX8 is a pair of box genes that are crucial for embryogenesis in the thyroid, Mullerian duct, and kidney/upper urinary tract (76), serving as a diagnostic marker for renal, Mullerian, and thyroid-origin tumors (77). Tahir et al. revealed that immunohistochemistry analysis of PAX8 and SOX17 (positive PAX8 and negative SOX17 expression) aids in the diagnosis of MLA (78). Our study demonstrated that *PAX2* and *PAX8* were more upregulated in MLAs than in conventional gynecologic tumors and were enriched in mesonephric tubule morphogenesis. Our findings validated their potential as differential diagnostic markers for MLA at the RNA level, consistent with previous studies. These pathways and genes hold potential as diagnostic and therapeutic markers for MLAs.

The TME plays a crucial role in tumor progression and treatment (79). The TME of MLA is not yet well understood. Our findings showed that CD8 T cells, B cells, natural killer cells, MHCI, and Th1 signature were downregulated, whereas Th2 signature and resting mast cells were upregulated in MLAs. Tumor-infiltrating CD8 (+) T cells and natural killer cells act as effector cells against tumor cells and are associated with better clinical outcomes (80). Th2 signature and resting mast cells contribute to the immunosuppressive state (81). The expression of BTLA significantly increased, while the expressions of LAG3, PDCD1LG2 and VSIR decreased significantly. BTLA (CD272) is one of the key factors regulating stimulatory and inhibitory signals in the immune response. It belongs to the CD28 superfamily and is mainly expressed in T and B lymphocytes, macrophages, and dendritic cells (82). The BTLA signal transduction recruits SHP-1/SHP-2 through the phosphorylation motifs of ITIMs, thereby negatively regulating the immune response (83). LAG-3 (also known as CD223) is an immune checkpoint receptor protein, which is mainly expressed on activated T cells, NK cells, B cells, and plasma cell dendritic cells (84). PDCD1LG2 participates in inducing immune tolerance under both physiological and pathological conditions (85). In immune cells, VISR is mainly expressed by myeloid cells (neutrophils, monocytes, macrophages and dendritic cells), and it is an important regulator of immune homeostasis and anti-tumor immunity (86). These results indicated that the TME of MLA is immunosuppressed. Our study reported results similar to those for nonmesonephric-like gynecologic tumors in TCGA database. Therefore, MLA lacks an antitumor immune environment, making immunotherapy potentially ineffective. The molecular grouping of *KRAS*-mutated MLA is of great interest. *SPOP* and *PIK3CA* are mutually exclusive and associated with different immune microenvironments. *PIK3CA* mutations were more enriched in the upregulated KRAS signaling pathway, interferon-γ response, and other immune response pathways. The E2F target pathway was enriched in the KRAS_*SPOP* mutation group. IFN-γ is critical in regulating immune responses, especially in malignant tumors (87). In MLAs patients with KRAS_*SPOP* mutations, the expression levels of CINSARC, GGI, and other markers related to cell proliferation were higher, indicating higher cell proliferation and malignancy in the KRAS_*SPOP mutation* group than in the KRAS_*PIK3CA mutation* group. A previous study revealed that *SPOP* mutations promote tumor immune escape through the IRF1–PD-L1 axis in endometrial cancer (26). Therefore, for patients with MLA who have both *KRAS* and *SPOP* mutations, more attention should be paid to subsequent treatment.

Owing to the limited sample size, the findings of this study, especially novel molecular and TME characteristics, need further validation. A larger cohort and wider multi-omics studies, encompassing noncoding RNA, protein, and methylation, are warranted to address the limitation.

In conclusion, our study showed that the significantly upregulated genes in MLA were mainly enriched in cell cycle and kidney development–related pathways. The TME of MLA is immunosuppressed, indicating that MLA is a cold tumor. In particular, MLA with both *KRAS* and *SPOP* mutations had a colder immune microenvironment.

## Data Availability

The original contributions presented in the study are included in the article/**Supplementary Material**. Further inquiries can be directed to the corresponding authors.

## Supplementary material

Supplementary Figure 1 Immunohistochemical staining of differential diagnostic markers in mesonephric-like adenocarcinomas (MLAs).

Supplementary Figure 2 Correlation analysis between baseline characteristics and survival. **(a, b)** The correlations of age, microsatellite instability (MSI), homologous recombination deficiency (HRD), tumor mutation burden (TMB), and clinical stage with progression-free survival (PFS) and overall survival (OS).

Supplementary Figure 3 Principal component analysis of mRNA, Functional Gene Expression (FGE), and HALLMARK pathways in mesonephric-like adenocarcinomas (MLAs) at three sites (cervical, ovary, and uterus). **(a)** Principal component analysis of mRNA expression in MLAs at three sites. **(b, c)** Single-sample gene set enrichment analysis (GSEA) of MLAs at three sites.

Supplementary Figure 4 the differences in the expression of fibroblast markers FAP and MFAP5 in MLA and normal tissues

Supplementary Figure 5 Differential expression of immune checkpoints in mesonephric-like adenocarcinomas (MLAs) and ovarian cancer (OV) **(a)**, cervical squamous cell carcinoma and endocervical adenocarcinoma (CESC) **(b)**, uterine corpus endometrial carcinoma (UCEC) **(c)** in The Cancer Genome Atlas (TCGA) database.

Supplementary Figure 6 The differences in immune characteristics between KRAS mutant MLA and KRAS mutant endometrial cancer in TCGA.

Supplementary Figure 7 Key genes enriched in mesonephric-like adenocarcinomas (MLAs) derived from the cervix **(a)**, ovary **(b)**, and uterus **(c)**.

Supplementary Figure 8 Differences in immune characteristics between mesonephric-like adenocarcinomas (MLAs) and ovarian cancer (OV) (a), cervical squamous cell carcinoma and endocervical adenocarcinoma (CESC) (b), and uterine corpus endometrial carcinoma (UCEC) (c) in The Cancer Genome Atlas (TCGA) database.

Supplementary Figure 9 Differences in immune characteristics among KRAS_PIK3CA, KRAS_SPOP, and other groups. **(a)** The differences in immune signatures among the three groups were compared via single-sample gene set enrichment analysis (GSEA). **(b)** Expression levels of IFN_10, IFN_18, IFN_6, STAT.signature, Teffector, and Teffector_IFN in the three groups. **(c)** The expression levels of proliferation-related markers (Complexity Index in Sarcomas [CINSARC], Core ESC-like Module, genomic grade index [GGI], and Sixteen_kinase) in the three groups. Sixteen_kinase refers to the kinase score of 16 genes encoding serine/threonine kinases involved in mitosis

Supplementary Figure 10 The differences in PFS and OS between the KRASmut/PIK3CAmut group and the KRASmut/SPOPmut group.
