# Supplementary figures and images for: Immune microenvironment heterogeneity characterizes biologically distinct KRASmut/SPOPmut and KRASmut/PIK3CAmut mesonephric-like adenocarcinoma subtypes revealed by integrated whole-exome and transcriptomic profiling
Source: Front Immunol. 2025 Jul 16;16:1605227. doi: 10.3389/fimmu.2025.1605227 (PMC12307467; doi:10.3389/fimmu.2025.1605227)

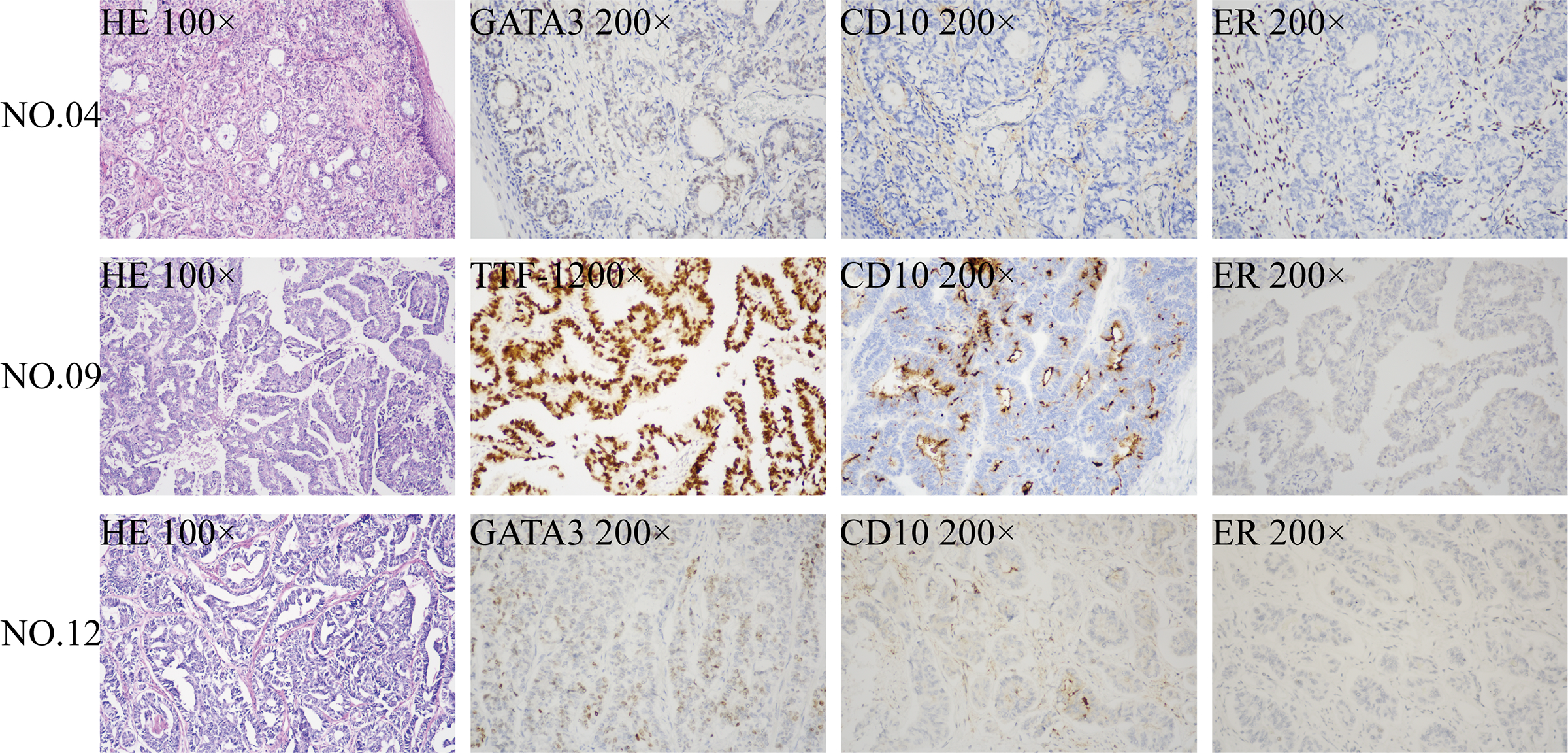

Supplement: Supplementary Figure 1 — Immunohistochemical staining of differential diagnostic markers in mesonephric-like adenocarcinomas (MLAs). [file Image1.tif]

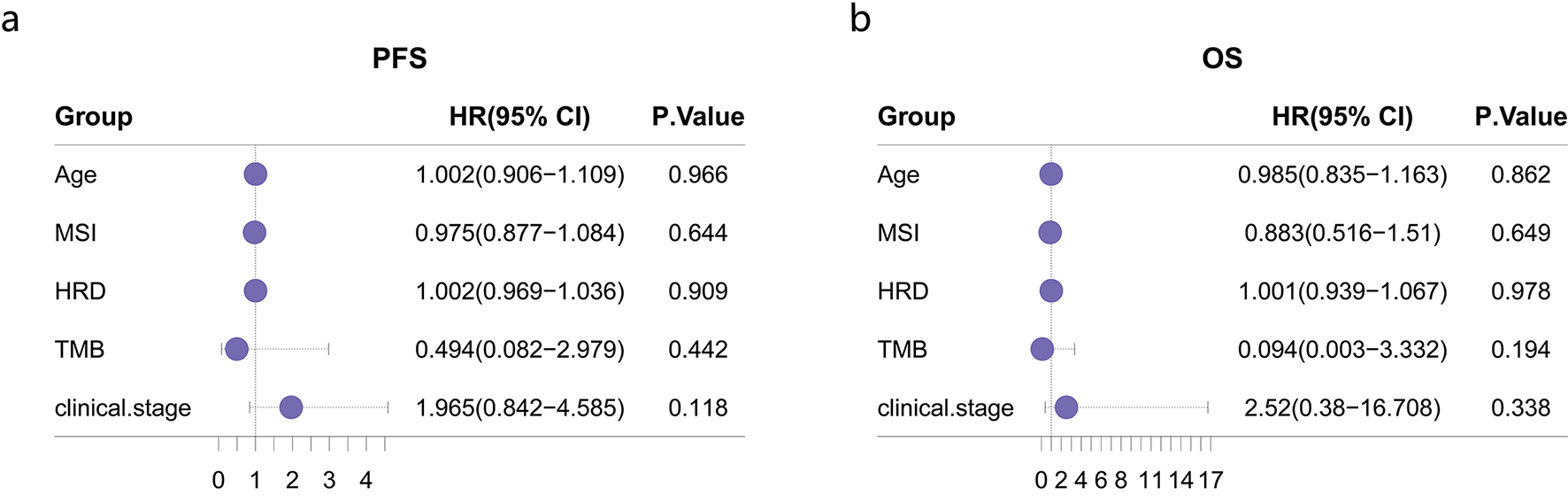

Supplement: Supplementary Figure 2 — Correlation analysis between baseline characteristics and survival. (a, b) The correlations of age, microsatellite instability (MSI), homologous recombination deficiency (HRD), tumor mutation burden (TMB), and clinical stage with progression-free survival (PFS) and overall survival (OS). [file Image2.tif]

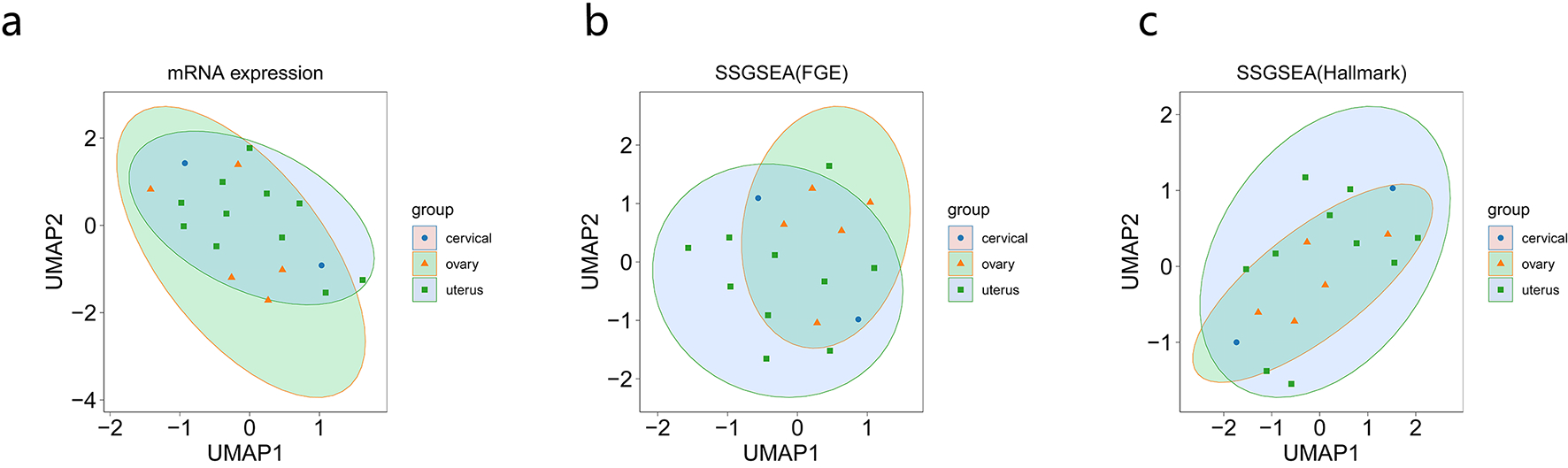

Supplement: Supplementary Figure 3 — Principal component analysis of mRNA, Functional Gene Expression (FGE), and HALLMARK pathways in mesonephric-like adenocarcinomas (MLAs) at three sites (cervical, ovary, and uterus). (a) Principal component analysis of mRNA expression in MLAs at three sites. (b, c) Single-sample gene set enrichment analysis (GSEA) of MLAs at three sites. [file Image3.tif]

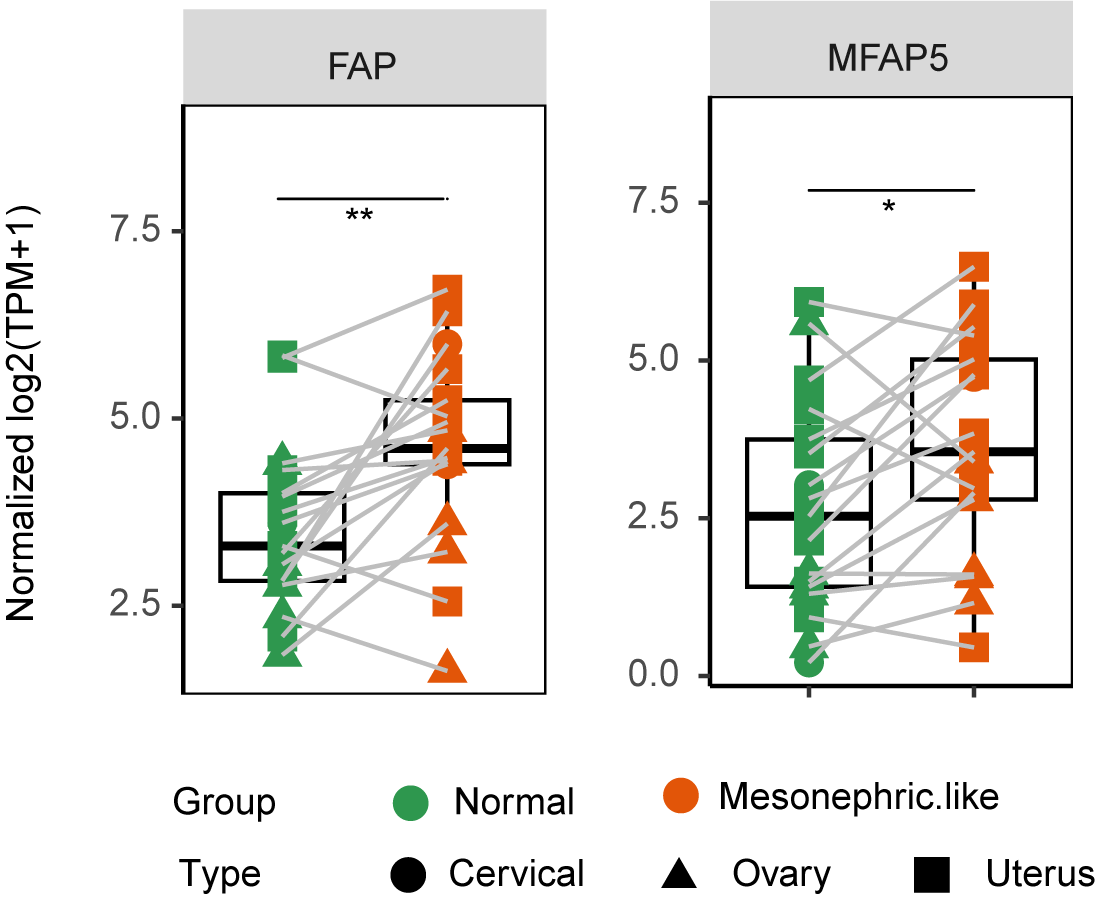

Supplement: Supplementary Figure 4 — the differences in the expression of fibroblast markers FAP and MFAP5 in MLA and normal tissues [file Image4.tif]

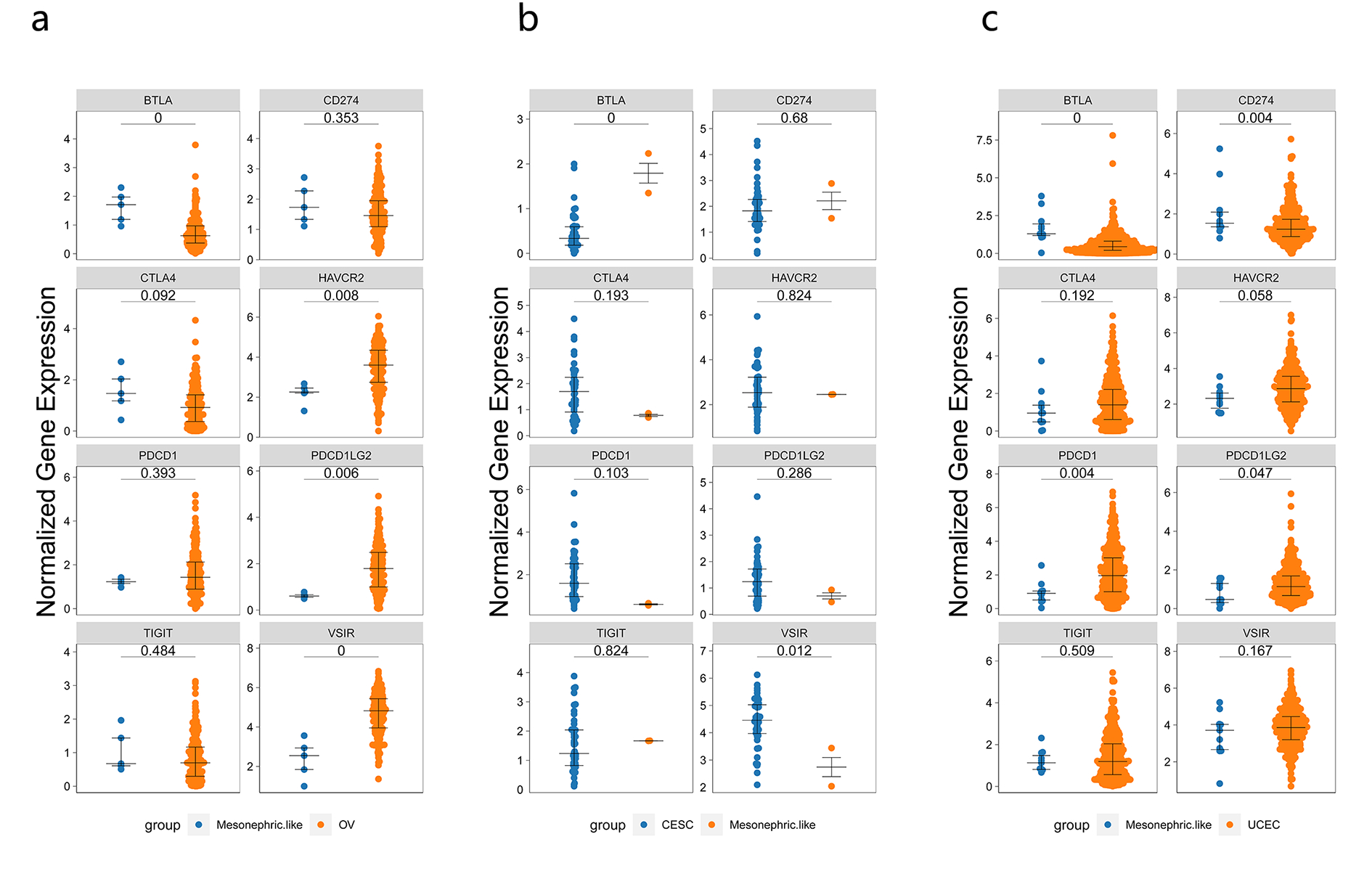

Supplement: Supplementary Figure 5 — Differential expression of immune checkpoints in mesonephric-like adenocarcinomas (MLAs) and ovarian cancer (OV) (a), cervical squamous cell carcinoma and endocervical adenocarcinoma (CESC) (b), uterine corpus endometrial carcinoma (UCEC) (c) in The Cancer Genome Atlas (TCGA) database. [file Image5.tif]

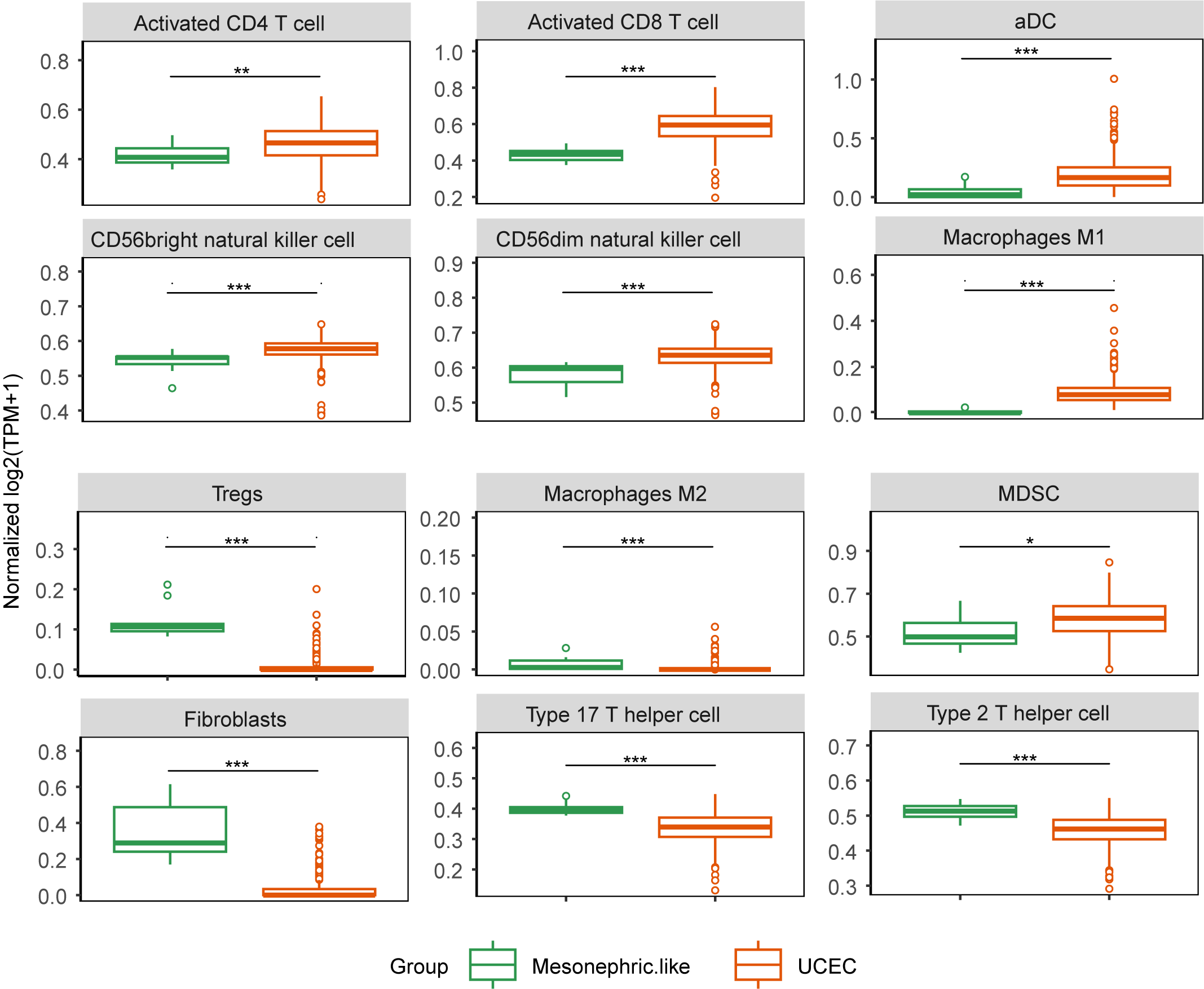

Supplement: Supplementary Figure 6 — The differences in immune characteristics between KRAS mutant MLA and KRAS mutant endometrial cancer in TCGA. [file Image6.tif]

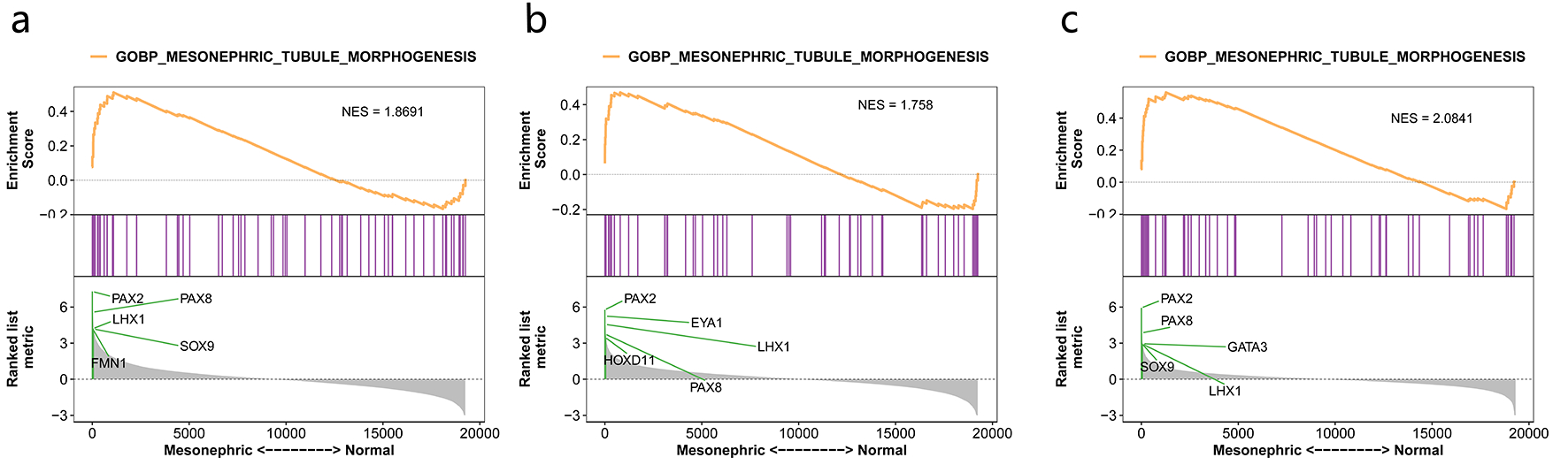

Supplement: Supplementary Figure 7 — Key genes enriched in mesonephric-like adenocarcinomas (MLAs) derived from the cervix (a), ovary (b), and uterus (c). [file Image7.tif]

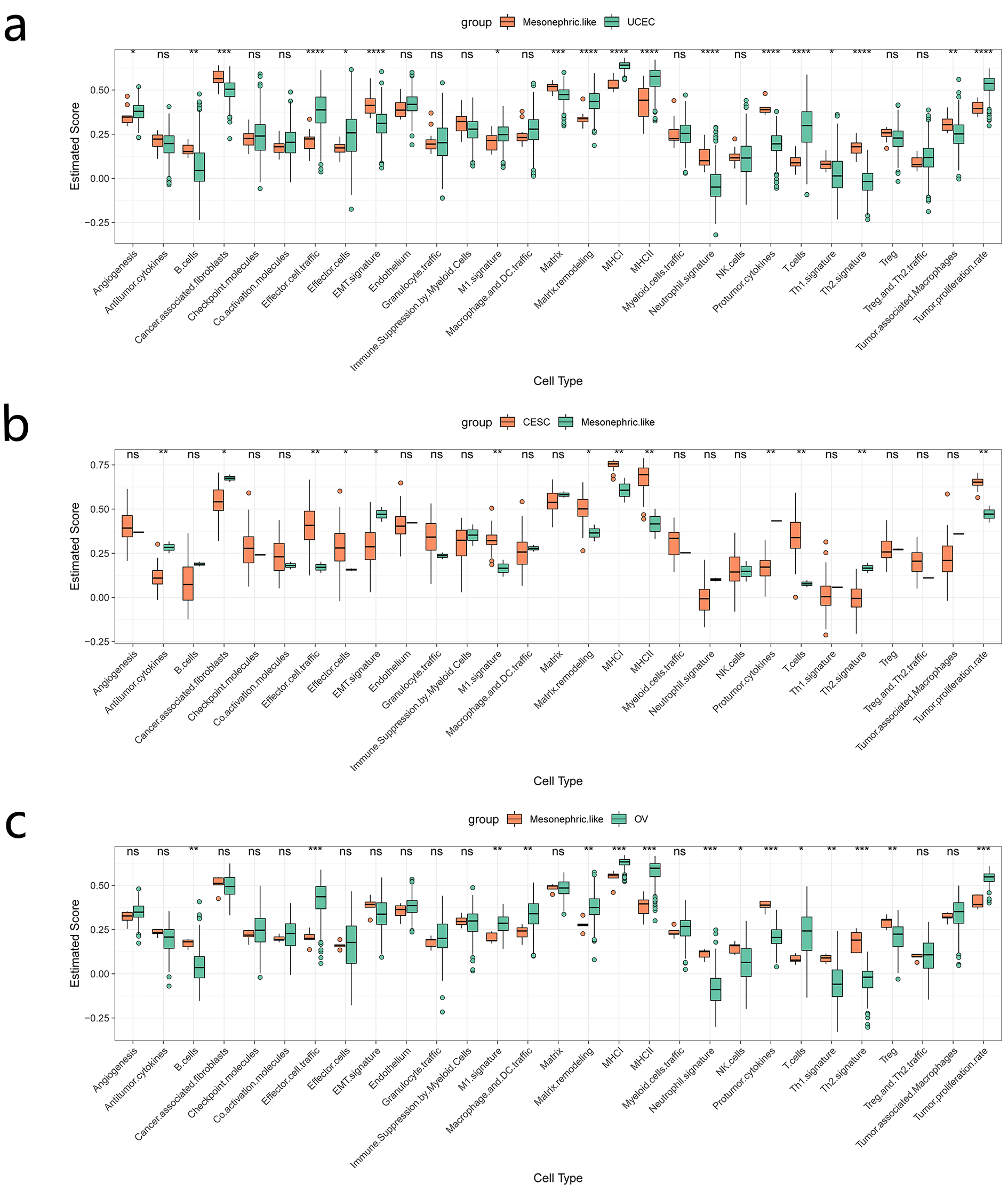

Supplement: Supplementary Figure 8 — Differences in immune characteristics between mesonephric-like adenocarcinomas (MLAs) and ovarian cancer (OV) (a), cervical squamous cell carcinoma and endocervical adenocarcinoma (CESC) (b), and uterine corpus endometrial carcinoma (UCEC) (c) in The Cancer Genome Atlas (TCGA) database. [file Image8.tif]

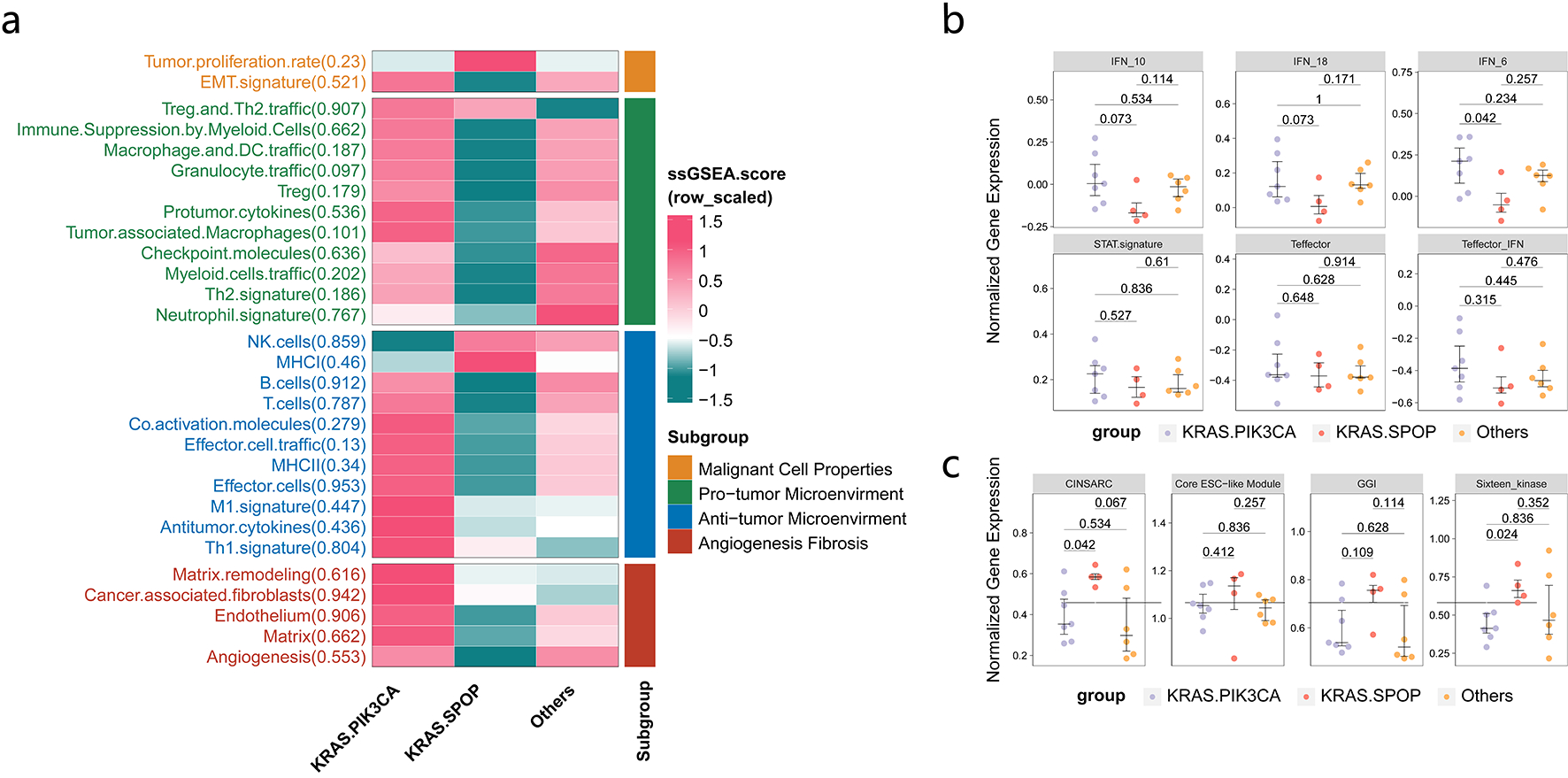

Supplement: Supplementary Figure 9 — Differences in immune characteristics among KRAS_PIK3CA, KRAS_SPOP, and other groups. (a) The differences in immune signatures among the three groups were compared via single-sample gene set enrichment analysis (GSEA). (b) Expression levels of IFN_10, IFN_18, IFN_6, STAT.signature, Teffector, and Teffector_IFN in the three groups. (c) The expression levels of proliferation-related markers (Complexity Index in Sarcomas [CINSARC], Core ESC-like Module, genomic grade index [GGI], and Sixteen_kinase) in the three groups. Sixteen_kinase refers to the kinase score of 16 genes encoding serine/threonine kinases involved in mitosis [file Image9.tif]

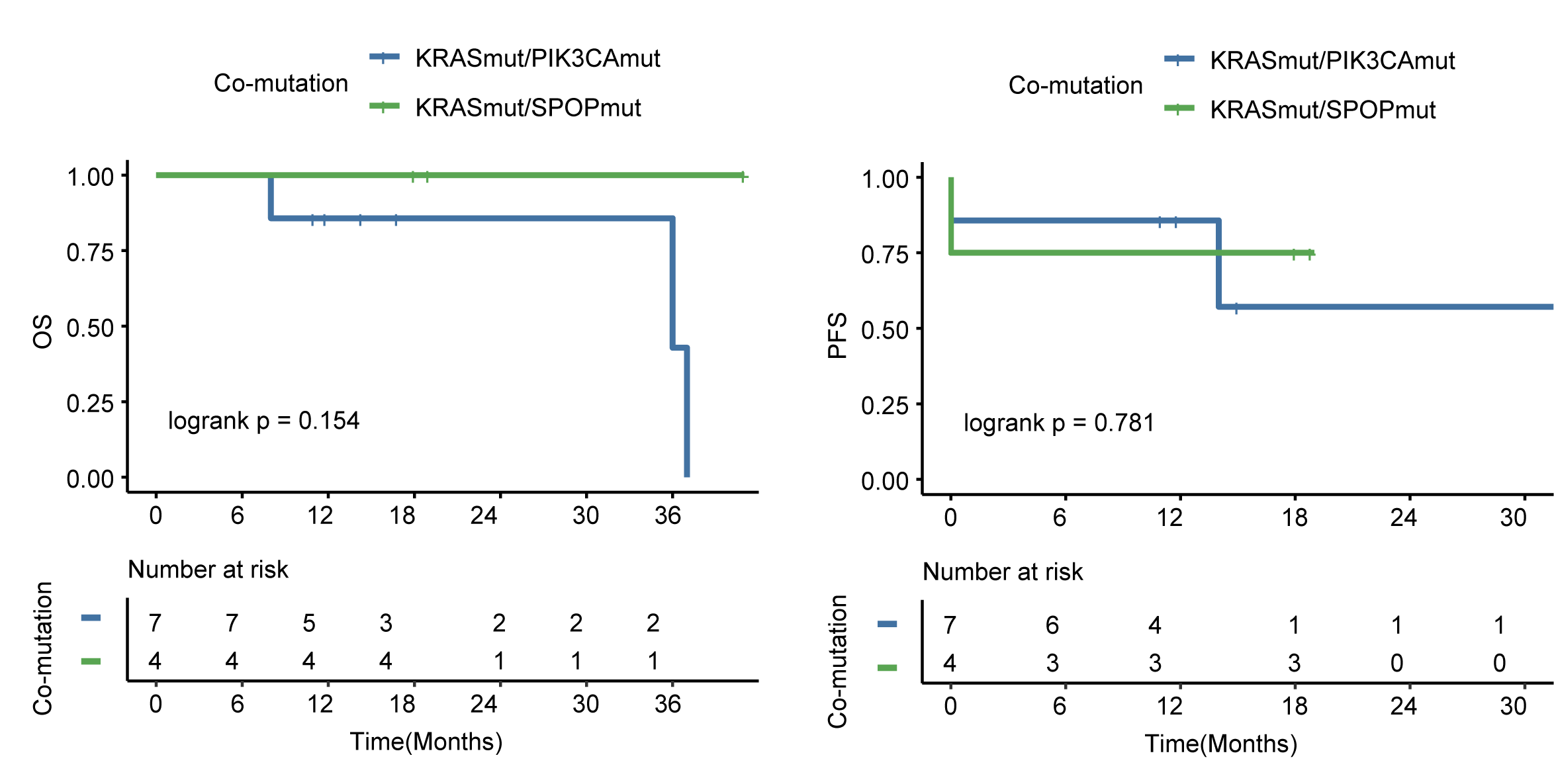

Supplement: Supplementary Figure 10 — The differences in PFS and OS between the KRASmut/PIK3CAmut group and the KRASmut/SPOPmut group. [file Image10.tif]
